# Plankton Community Respiration and Particulate Organic Carbon in the Kuroshio East of Taiwan

**DOI:** 10.3390/plants11212909

**Published:** 2022-10-29

**Authors:** Chung-Chi Chen, Pei-Jie Meng, Chih-hao Hsieh, Sen Jan

**Affiliations:** 1 Department of Life Science, National Taiwan Normal University, 88, Sec. 4, Ting-Chou Road, Taipei 11677, Taiwan; 2 Graduate Institute of Marine Biology, National Dong Hwa University, Checheng, Pingtung 94450, Taiwan; 3 General Education Center, National Dong Hwa University, Shoufeng, Hualien 97401, Taiwan; 4 National Museum of Marine Biology and Aquarium, Checheng, Pingtung 94450, Taiwan; 5 Institute of Oceanography, National Taiwan University, Taipei 10617, Taiwan

**Keywords:** dissolved inorganic nutrients, particulate organic carbon, picoeukaryotes, picoplankton, plankton community respiration, *Prochlorococcus*, *Synechococcus*, the Kuroshio

## Abstract

Biological organic carbon production and consumption play a fundamental role in the understanding of organic carbon cycling in oceans. However, studies on them in the Kuroshio, the western boundary current in the North Pacific Ocean, are scarce. To better understand the variations of plankton community respiration (CR) and particulate organic carbon (POC), eight cruises. which covered four seasons over a 2-year period, were surveyed across the Kuroshio at the KTV1 transect east of Taiwan. Spatially, a coastal uplift of isotherms (i.e., onshore lifting and offshore deepening) was observed along the KTV1 transect. During the uplift, the cold and nutrient-rich deep waters shoal to shallow water and enhance phytoplankton growth, resulting in higher values of phytoplankton, POC, and plankton CR on the onshore side. In this study, phytoplankton was dominated by picophytoplankton including *Prochlorococcus*, *Synechococcus*, and picoeukaryotes. Plankton CR was low, and its mean depth-normalized integrated rate (the upper 100 m water depth) ranged from 7.07 to 22.27 mg C m^−3^ d^−1^, to which the picophytoplankton and heterotrophic bacteria contributed the most. The mean depth-normalized integrated value of POC ranged from 12.7 to 21.6 μg C L^−1^. POC is mainly associated with phytoplankton biomass with a mean carbon ratio of chlorophyll *a*/POC ≈ 1.03. All results suggest that plankton CR and POC variations may be associated with picoplankton dynamics in the Kuroshio.

## 1. Introduction

Biological carbon production and consumption play an important role in influencing the biological pump and global carbon cycle of marine systems [1,2]. In the oligotrophic ocean, phytoplankton produces organic carbon through primary production in the euphotic zone, and particulate organic carbon (POC) is conventionally treated as a proxy of phytoplankton [3]. It can provide food to nourish nekton and thus may play an important role in the marine food web. Although a large proportion of marine primary production is remineralized, POC produced in the euphotic zone is the main form of the biological pump [4,5]. It can be transported to deep water mainly in the form of fast-sinking particulate matter and slower marine snow or aggregate floc to bury and reserve carbon in the deep ocean [6,7]. As for remineralization, plankton community respiration (CR) plays a major role in pelagic ecosystems [8,9]. Therefore, the characterization of plankton CR and POC is a fundamental requirement for understanding the organic carbon cycle in the ocean.

The Kuroshio is the western boundary current in the North Pacific Ocean. It originates from the North Equatorial Current and flows along the east coast of Taiwan before reaching the southeast coast of Japan [10]. Kuroshio water (KW) is oligotrophic and has low biological productivity [11]. In spite of the oligotrophy, the Kuroshio supports a high abundance of fish (e.g., [12,13]). This inconsistency is known as the “Kuroshio paradox” [14]. To nourish nekton, it is important to evaluate the essential food sources (i.e., primary productivity) in the pelagic ecosystem. In the Kuroshio, high primary productivity has been found in several regions that are mostly enhanced by bringing nutrient-rich subsurface KW to the surface (e.g., [15,16,17,18]). In this oligotrophic ocean, picophytoplankton dominates the phytoplankton [15]. Despite their small size, autotrophic picoplankton has been shown to be an important POC flux in the surface layer over large oceanic regions [19]. In the Kuroshio, POC is primarily of biological origin, and in situ production is the dominant process for controlling POC loading in the euphotic zone [20]. However, its biological components have not been examined. POC can also serve as a fundamental nutrition source for higher trophic levels (e.g., nekton), although it is surprising that data on this in the Kuroshio are scarce (e.g., [20,21]). As for organic carbon consumption (i.e., plankton CR), an even rarer dataset has been reported in the Kuroshio and the western Pacific Ocean [22,23,24].

**Figure 1:**
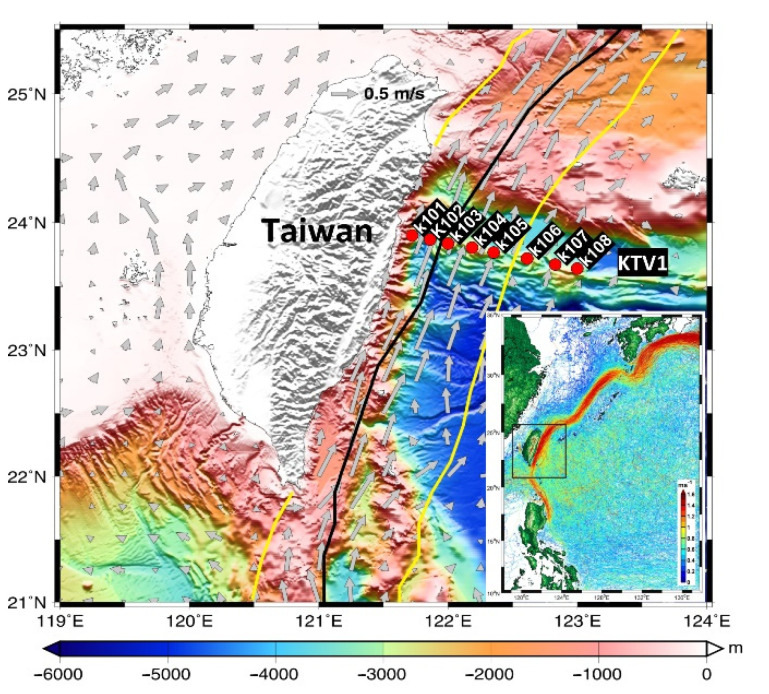
Sampling stations (K101–K108) along the KTV1 transect of the Kuroshio east of Taiwan. The climatological current velocity (gray arrows) at a depth of 30 m was computed from the historical ADCP dataset available from Taiwan’s Ocean Data Bank. The bold black line indicates the maximum velocity axis at 15 m depth obtained from the surface drifter data. The solid yellow line indicates the 0.2 m s^−1^ isotach at 30 m depth obtained from the historical ADCP dataset [28]. The path of the Kuroshio with velocity >1 m s^−1^ is indicated by surface drifter trajectories and speeds (data available at http://www.coriolis.eu.org/Data-Products/Data-Delivery/Data-selection; accessed on 27 February 2018) in the lower right inset, where the black dotted rectangle marks the map area. (Modified from Figure 1 of Chen et al. [15]).

Furthermore, a coastal uplift has been observed in this study region in the Kuroshio east of Taiwan [15]. During the uplift, the cold and nutrient-rich deep waters shoal to shallow water and enhance phytoplankton growth [15,25,26]. The shoaled nutrient-rich waters may be important for biological production, consumption, and plankton dynamics (e.g., picoplankton composition) in oligotrophic ecosystems such as the Kuroshio [27]; however, how they affect and contribute to POC and plankton CR has not been evaluated. This study therefore intends to reveal the seasonal and spatial variations of POC and plankton CR in the Kuroshio ecosystem, including its biological components, especially off the east coast of Taiwan. For this purpose, data were applied from eight cruises covering different seasons over a 2-year period with stations across the Kuroshio east of Taiwan (Figure 1). Noticeably, there are no large rivers in the nearby region of this study area.

## 2. Results and Discussion

### 2.1. Spatial and Temporal Hydrographic Pattern in the Kuroshio

**Figure 2:**
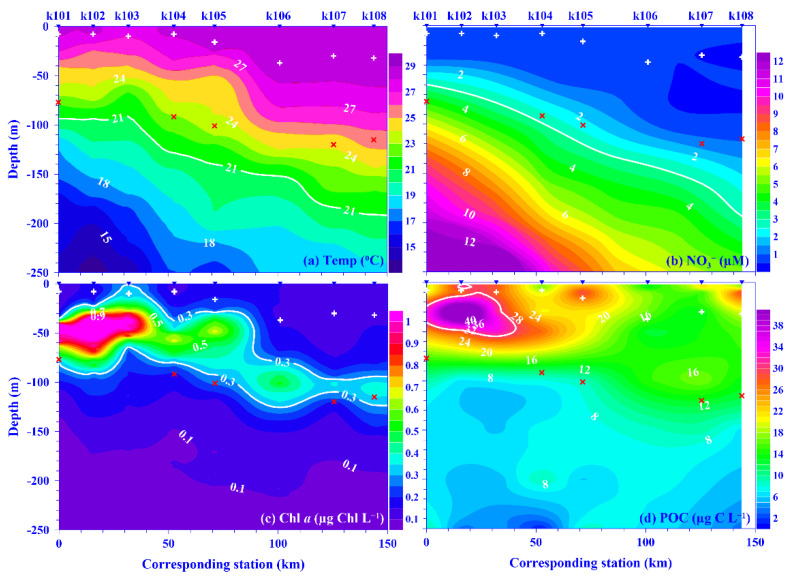
Typical depth profile contour plots of (**a**) temperature (Temp; °C), (**b**) nitrate (NO_3_^−^; µM), (**c**) chlorophyll *a* (Chl *a*; µg Chl L^−1^), and (**d**) particulate organic carbon (POC; µg C L^−1^) within 250 m water depth along the KTV1 transect in September 2015. Contour lines in white bold are guidelines, including temperature (21 °C), nitrate (1 µM), Chl *a* (0.3 µg Chl L^−1^), and POC (30 µg C L^−1^). The inverse triangles at the top of each panel indicate the locations of stations k101–k108 along the KTV1 transect. Corresponding distance (=0) start from St. k101. For reference, the depths of mixed layer (+) and euphotic zone (×) are also marked.

High salinity and temperature are typical characteristics of KW. This phenomenon has also been observed in the Kuroshio east of Taiwan. The mean values (±standard deviation [SD] for this and all parameters discussed henceforth) of temperature and salinity in the surface water were in the range of 22.5 (±0.3) to 30.2 (±0.8) °C and 34.08 (±0.13) to 34.79 (±0.05), respectively, over each sampling period. Seasonally, the surface temperature was higher in the summer compared with other seasons, but this trend was not observed in the surface water salinity (Figure S1). Spatially, the temperature and salinity of the surface water were similar across the Kuroshio at the KTV1 transect east of Taiwan (Figure 1). Vertically, the seawater temperature displayed a coastal uplift (i.e., onshore lifting and offshore deepening) of the isotherms along the KTV1 transect, using data from September 2015 as a typical example (Figure 2a). It has been suggested that this coastal uplift phenomenon occurs year-round in a broad-scale region on the onshore side of the Kuroshio east of Taiwan [15,29]. As for the mixed layer (M_D_), it was shallower in the summer and deeper in other seasons, and its average value ranged from 12.9 (±3.9) to 96.5 (±47.0) m over each sampling period. Spatially, the M_D_ depth was deeper at offshore stations compared to onshore stations (Table S1). The euphotic zone (Z_E_) was, however, more consistent spatiotemporally (Table S1), with an average depth of 104.6 (±12.3) m, and the Z_E_ value seemed consistent in the Kuroshio east of Taiwan [15,30]. This suggests that light is not a limiting factor for phytoplankton growth, spatiotemporally, in this subtropical region.

**Figure 3:**
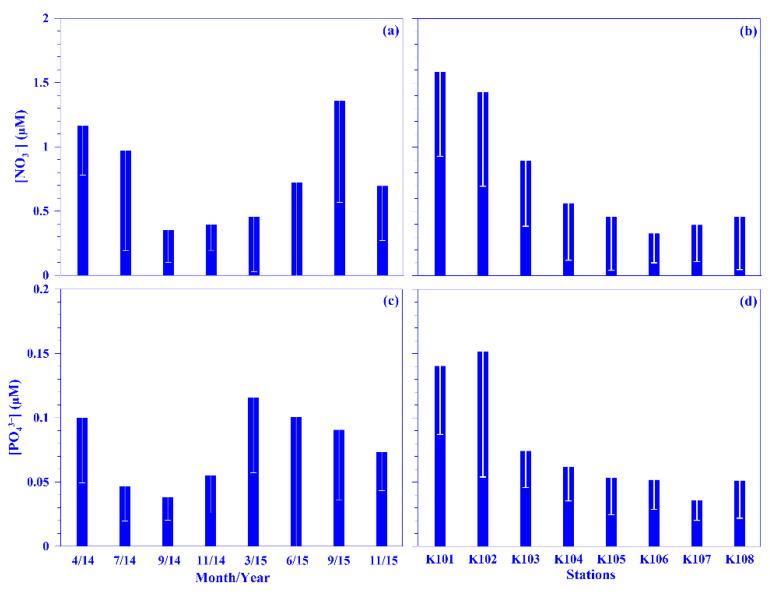
Temporal and spatial variations of the mean values of nitrate (NO_3_^−^; (**a**,**b**)) and phosphate (PO_4_^3−^; (**c**,**d**)) across the KTV1 transect of the Kuroshio. The depth-normalized integrated value over 100 m water column of each variable at each sampling station was used for the mean values. The mean values were averaged across all stations of the KTV1 transect for each sampling period or across each station of the KTV1 transect for all sampling periods. The standard deviations are illustrated as vertical white lines with caps.

Even though the nutrient concentration in the surface water was low in the oligotrophic Kuroshio [31,32], its values were occasionally high in this study. On average, the values for nitrate and phosphate in the surface water were in the range of 0.05 (±0.01) in November 2015 to 0.98 (±0.26) μM in April 2014 and 0.02 (±0.00) in September 2014 to 0.11 (±0.07) μM in April 2014, respectively, over each sampling period. Spatiotemporally, the nutrient concentrations in the surface water showed no distinct distribution pattern across the Kuroshio at the KTV1 transect, eastern Taiwan. The mean depth-normalized integrated (i.e., the upper 100 m water depth; see Materials and Methods for details) nutrient concentrations were in the range of 0.35 (±0.25) to 1.36 (±0.79) μM for nitrate and 0.04 (±0.02) to 0.12 (±0.06) μM for phosphate over each sampling period (Figure 3a,c). Temporally, there was no seasonal trend for the mean depth-normalized integrated values of nutrients (both nitrate and phosphate; Figure 3a, c); however, a distinct spatial distribution pattern was observed for those values; for example, they were over two times higher in the onshore stations (e.g., NO_3_^−^ = 1.12 μM; PO_4_^3−^ = 0.11 μM) than that in the offshore stations (e.g., NO_3_^−^ = 0.41 μM; PO_4_^3−^ = 0.05 μM) across the KTV1 transect (Figure 3b,d). Vertically, corresponding to the isotherms uplifting, the nitrate concentration contours also demonstrated a coastal uplift pattern along the KTV1 transect (Figure 2b). The higher mean depth-normalized integrated nutrient value in the onshore stations was mainly attributed to the coastal uplift that shoals the cold, nutrient-rich deep water to the shallow water. This assumption could be evidenced significantly by the positive linear relationships between nutrients (nitrate or phosphate) and water density, using all pooled data in this study. The linear trend was also statistically significant for nutrients and water temperature, albeit with negative slopes (all *p* < 0.001; data not shown). The uplifted nutrients may thereafter enhance primary production or change plankton composition in the Kuroshio ecosystem [27,33]. Both dissolved inorganic nutrient concentration/availability and the nitrate-to-phosphate (N/P) molar ratio have important impacts on plankton dynamics [34]. The N/P molar ratio was in the range of 9.3–29.5 over each sampling period, with an average value of 12.2 (Figure S2). This average ratio is consistent with the low N/P ratio (e.g., <16) observed in the KW [31]. Noticeably, it varied temporally, and this might result in variant responses on plankton dynamics.

### 2.2. Spatial and Temporal Variations of Plankton CR and POC in the Kuroshio

**Figure 4:**
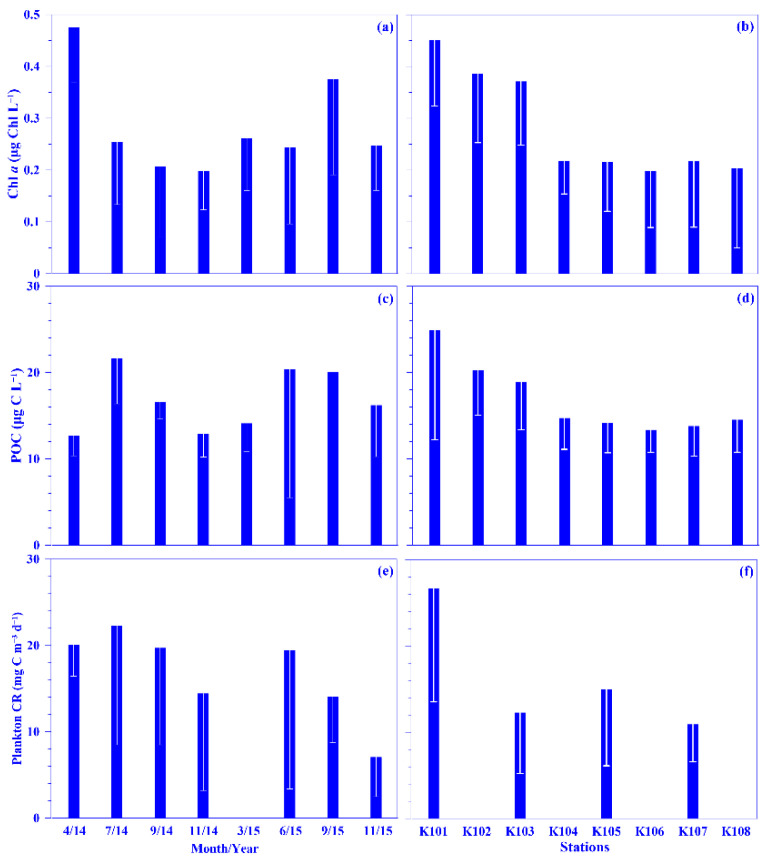
Temporal and spatial variations of the mean values of chlorophyll *a* (Chl *a*; (**a**,**b**)), particulate organic carbon (POC; (**c**,**d**)), and plankton community respiration (Plankton CR; (**e**,**f**)) across the KTV1 transect of the Kuroshio. The depth-normalized integrated value over 100 m water column of each variable at each sampling station was used for the mean values. The mean values were averaged across all stations of the KTV1 transect for each sampling period or across each station of the KTV1 transect for all sampling periods. The standard deviations are illustrated as vertical white lines with caps.

**Figure 5:**
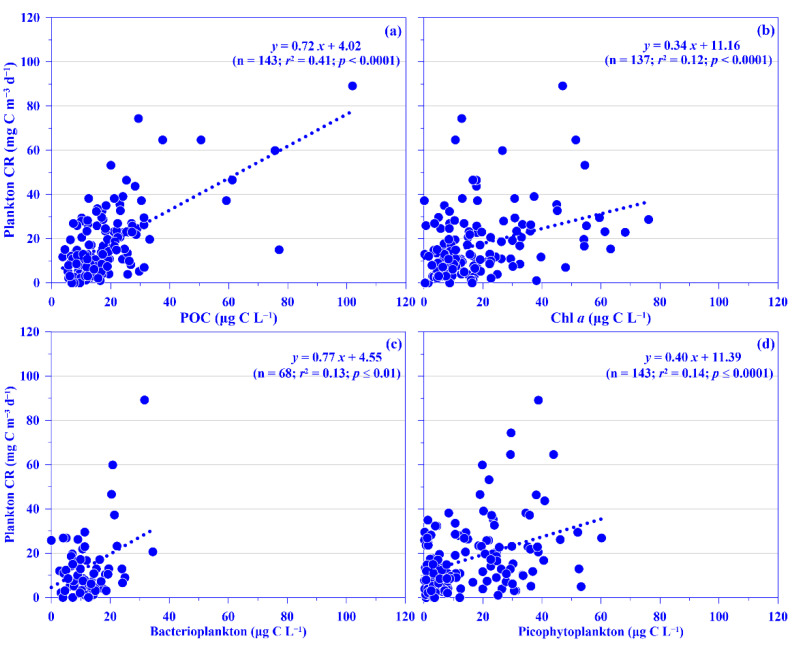
Relationships between concentration of plankton community respiration (plankton CR) and (**a**) particulate organic carbon (POC), (**b**) chlorophyll *a* (Chl *a*), (**c**) heterotrophic bacterioplankton, and (**d**) all picophytoplankton (i.e., *Prochlorococcus* + *Synechococcus* + picoeukaryotes) for all pooled data. Note that the variables are in carbon units. The *r*^2^ and *p* values of linear relationships are shown.

Plankton CR plays a major role in the ocean by consuming organic carbon. Datasets on this are, however, rare compared with those on global primary production, since acquiring them is labor intensive [9], and they are even more scarce in the Kuroshio [22,23,24]. In this study, the mean depth-normalized integrated (i.e., the upper 100 m water depth) rate of plankton CR ranged from 7.07 (±4.57) to 22.27 (±13.76) mg C m^−3^ d^−1^ over each sampling period, which places the values at the lower end of reported plankton CR in the open ocean, which with a median value of 31.50 mg C m^−3^ d^−1^ [8]. No temporal patterns were observed in the mean plankton CR rate, although the rate seemed lower during the winter period (Figure 4e). Spatially, a higher rate (26.62 ±13.11 mg C m^−3^ d^−1^) was measured in the onshore station (k101; Figure 4f); this rate is comparable to that observed in upwelling northeast of Taiwan (C.-C. Chen’s unpublished data). Vertically, in general, the plankton CR rate increased from the surface water to 30 water depth and then decreased down to the bottom of the euphotic depth, i.e., ca. 100 m water depth in this study (Figure S3). This spatial distribution pattern may be associated with phytoplankton biomass (used chlorophyll *a* [Chl *a*] proxy), with its growth enhanced by the coastal uplift nutrient (see below for details). Plankton CR was significantly and linearly related to concentrations of POC or Chl *a*, using all pooled data (all *p* < 0.0001; Figure 5a,b) or using all the depth-normalized integrated values at each sampling station (all *p* < 0.05; data not shown). This phenomenon has also been observed frequently in the oligotrophic ocean [9]. Further analysis showed a significant linear relationship between plankton CR and the biomass of heterotrophic bacterioplankton or total picophytoplankton, using all pooled data (all *p* < 0.0001; Figure 5c,d). In addition, Plankton CR was significant in multiple linear regression with picophytoplankton biomass (i.e., *Prochlorococcus*, *Synechococcus*, and picoeukaryotes; *p* < 0.001). All the results suggest that the plankton CR may be mostly attributed to phytoplankton, and especially picophytoplankton and heterotrophic bacterioplankton, in the Kuroshio ecosystem east of Taiwan.

**Figure 6:**
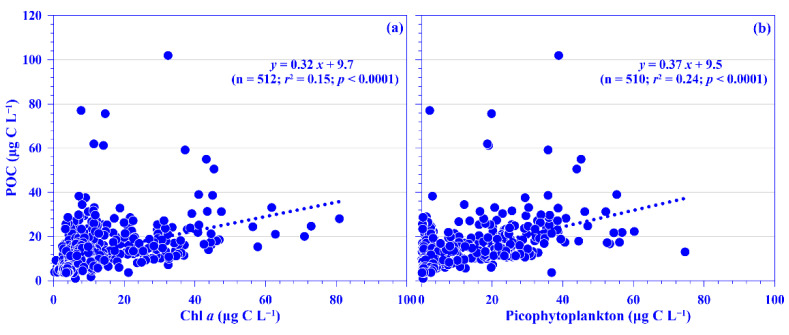
Relationships between concentration of particulate organic carbon (POC) and (**a**) chlorophyll *a* (Chl *a*) and (**b**) picophytoplankton for all pooled data. The *r*^2^ and *p* values of linear relationships are shown.

POC is normally treated as a proxy for phytoplankton in the oligotrophic ocean, such as in the Kuroshio east of Taiwan [3,20,21]. In this study, the lowest and highest POC concentrations were 1.8 and 77.1 μg C L^−1^, respectively, with mean depth-normalized integrated values ranging from 12.7 (±2.3) to 21.6 (±5.2) μg C L^−1^ (Figure 4c). The concentration was in the range of the reported value, 2–99 μg C L^−1^, in the KW [35,36,37,38]. Notably, the POC value is relatively low in the KW compared to that in the East China Sea (ECS) shelf ecosystem; for example, its mean value over the entire ECS shelf was 227.5 (±141.9) μg C L^−1^ [39]. Spatially and temporally, distinct distribution patterns were observed for the mean POC values in the surface water and its depth-normalized integrated value. Seasonally, its values were higher in the summer and autumn, and spatially, it showed a higher value in the onshore stations and a lower value in the offshore stations across the KTV1 transect (Figure 2d and Figure 4c,d). This spatial distribution pattern may be associated with phytoplankton distribution (see below for details). Vertically, the POC concentration demonstrated a decreasing trend from the surface water down to the deeper water column (250 m; Figure S4). This vertically decreasing trend was also observed in the mainstream of the Kuroshio east of Taiwan [37]. In addition, a linear relationship was significantly evidenced between concentrations of POC and Chl *a*, using all pooled data in this study (*p* < 0.0001; Figure 6a) or using all the depth-normalized integrated values at each sampling station (all *p* < 0.001; data not shown). This significant linear relationship has also been evidenced in the Kuroshio, and it is therefore suggested that POC in the euphotic layer is primarily of biological origin [20]. On average, the carbon ratio of Chl *a* to POC was 102.6 (±83.5)% using all data in this study. This suggests that the POC is mainly attributed to phytoplankton biomass in the oligotrophic KW [21]. In the oligotrophic ocean (e.g., the Kuroshio), phytoplankton is dominated by picophytoplankton [15,33]. Low POC export (or biological carbon pump) efficiencies have been observed in the western North Pacific Ocean, which suggests that these low efficiencies may be associated with the dominance of smaller particles and the process of degradation and subsequent remineralization of these small particles in the euphotic zone of the oligotrophic ocean [40]. Although POC flux was not measured in this study, a significant linear relationship was statistically evidenced between the POC concentration and total picophytoplankton biomass using all the pooled data (all *p* < 0.0001; Figure 6b). Autotrophic picoplankton has been shown to be an important POC flux in the surface layer over large oceanic regions [19].

### 2.3. Biological Variables Associated with Plankton CR and POC in the Kuroshio

A couple of biological variables may contribute to plankton CR and POC in the oligotrophic ocean, and this study focuses on the major contributors, including phytoplankton and heterotrophic bacterioplankton. For phytoplankton biomass, its mean value in the surface water ranged from 0.07 (±0.03) to 0.52 (±0.13) μg Chl L^−1^ (or 4.61 [±1.98] to 34.27 [±8.57] μg C L^−1^) over each sampling period. The mean depth-normalized integrated Chl *a* value was in the range of 0.20 (±0.08) to 0.48 (±0.11) μg Chl L^−1^ (or 13.18 [±5.27] to 31.63 [±7.25] μg C L^−1^) over each sampling period (Figure 4a). There was no seasonal trend for the mean Chl *a* concentration in the surface water or the depth-normalized integrated values (Figure 4a). Spatially, the depth-normalized integrated Chl *a* values were higher in the onshore and lower in the offshore stations, similar to other variables (Figure 4b). The higher phytoplankton biomass and subsurface Chl *a* maximum located shallower in the onshore might be enhanced by the coastal uplift nutrients. This phenomenon was also identified in our previous study [15]. The subsurface Chl *a* maximum value (e.g., 1.74 μg Chl L^−1^) was higher and located shallower onshore compared with that offshore in the KTV1 (Figure 2c). The assumption could be indirectly supported by a significant linear relationship evidenced between the values of Chl *a* and nutrients (e.g., nitrate or phosphate) by using all pooled data in this study (all *p* < 0.01). This result also suggests that phytoplankton growth may be limited by nutrient availability in the oligotrophic KW [15,33], but its growth may not be limited by light intensity, as identified in previous discussion.

In addition, the Chl *a* concentration was also significantly and linearly related to total autotrophic picoplankton (*Prochlorococcus* + *Synechococcus* + picoeukaryotes) in terms of abundance or biomass, using all pooled data in this study (all *p* < 0.001; Figure S5). This suggests that phytoplankton is as dominated by picophytoplankton in the study region as it is in the oligotrophic ocean (e.g., [41,42]). This assumption can be indirectly evidenced by the large value of the average biomass ratio of picophytoplankton to Chl *a* (92.8% [±129.8]), using all pooled data in this study. It also suggests that plankton CR and POC may contribute to picophytoplankton and dominate in the KW east of Taiwan. As stated above, this assumption can be indirectly supported by the significant linear relationship evidenced between plankton CR or POC concentration and total picophytoplankton biomass, using all pooled data (all *p* < 0.0001; Figure 5d and Figure 6b). Notably, in addition to picoplankton, Chl *a* and POC may also contribute to other size classes of plankton in the oligotrophic ocean [42]. For example, nano- and micro-phytoplankton have been estimated and contributed approximately 49.3% of Chl *a* concentration in the downstream area (ca., 100 km) of this study region (C.-h. Hsieh’s unpublished data). The size-fractioned analyses of Chl *a* and POC were, however, not measured in this study.

**Figure 7:**
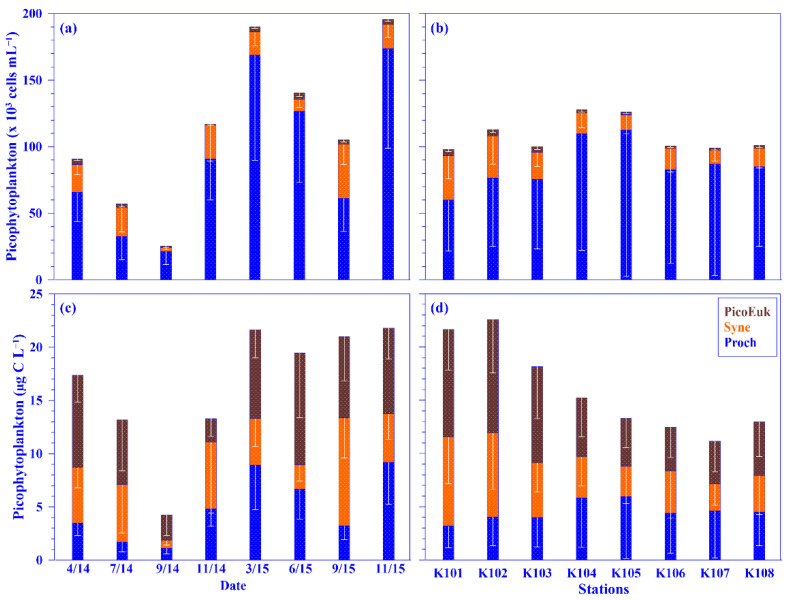
Temporal and spatial variations of the mean values of picophytoplankton (i.e., *Prochlorococcus* [Proch] + *Synechococcus* [Syne] + picoeukaryotes [PicoEuk]) in terms of abundance (**a**,**b**) and biomass (**c**,**d**) across the KTV1 transect of the Kuroshio. The depth-normalized integrated value over 100 m water column of each variable at each sampling station was used for the mean values. The mean values were averaged across all stations of the KTV1 transect for each sampling period or across each station of the KTV1 transect for all the sampling periods. The standard deviations are illustrated as vertical white lines with caps.

As for picophytoplankton, the mean depth-normalized integrated values of *Prochlorococcus*, *Synechococcus*, and picoeukaryotes were in the range of 21.34 (±9.96) to 173.61 (±74.67) × 10^3^ cells mL^−1^, 2.87 (±1.71) to 40.56 (±15.09) × 10^3^ cells mL^−1^, and 1.05 (±0.80) to 5.00 (±2.90) × 10^3^ cells mL^−1^ for abundance and of 1.13 (±0.53) to 9.20 (±3.96) μg C L^−1^, 0.72 (±0.43) to 10.14 (±3.77) μg C L^−1^, and 2.21 (±1.68) to 10.49 (±6.08) μg C L^−1^ for biomass, respectively, over each sampling period. The mean relative abundance of picophytoplankton was dominated by *Prochlorococcus* (77.1% ±13.5), followed by *Synechococcus* (19.6% ±12.9) and then picoeukaryotes (3.3% ±1.5); however, in terms of biomass, its mean value was approximately similar in *Prochlorococcus* (4.91 ±3.10 μg C L^−1^) and *Synechococcus* (4.86 ±2.79 μg C L^−1^) but was slightly higher in picoeukaryotes (6.74 ±3.00 μg C L^−1^), using all pooled data. Similar dominance of picoplankton abundance, namely *Prochlorococcus* and *Synechococcus*, was also observed in the KW and the subtropical northwest Pacific [15,33]. Even though the abundance of picoeukaryotes was low, its biomass was higher than that of *Prochlorococcus* or *Synechococcus* in this study due to the larger carbon conversion factor for picoeukaryotes (see Materials and Methods). Temporally, no seasonal trend was observed for picophytoplankton; however, spatially, a distinct pattern was observed in the distribution of *Synechococcus* and picoeukaryotes—that is, its values, in terms of abundance and biomass, were higher in onshore stations and lower in offshore stations across the KTV1 transect (Figure 7). This spatial distribution pattern may be associated with the suggestion that the growth of *Synechococcus* and picoeukaryotes in onshore stations may be enhanced by the onshore uplift nutrients, according to previous research [15]. The enhancement of growth of *Synechococcus* and picoeukaryotes by nutrient concentrations has also been observed in other oligotrophic oceans [43]. Notably, a linear relationship was significantly evidenced between an abundance of picophytoplankton (i.e., *Prochlorococcus*, *Synechococcus*, or picoeukaryotes) and nutrient (nitrate or phosphate) concentrations, using all pooled data (all *p* < 0.001). This result indirectly supports that the growth of picophytoplankton is associated with nutrient concentrations/availability [15,43]. Additionally, the growth of picophytoplankton, and especially *Prochlorococcus*, may also be associated with the N/P molar ratio and/or water temperature, as observed in the KW [15,33].

In addition to phytoplankton, heterotrophic bacterioplankton is one of the major contributors to plankton CR in the open ocean [9]. In this study, the abundance of heterotrophic bacterioplankton was only measured in the 2015 cruises; its mean depth-normalized integrated biomass was in the range of 8.07 (±1.94) to 16.75 (±3.03) μg C L^−1^ (or 4.03 [±0.97] to 8.38 [±1.52] × 10^5^ cells mL^−1^ in abundance) over each sampling period in 2015. No distinct pattern, spatially or temporally, was observed in the distribution of the heterotrophic bacterioplankton (Figure S6). Further analysis showed there to be a significant linear relationship between abundance (or carbon biomass) of heterotrophic bacterioplankton versus *Prochlorococcus*, picoeukaryotes, or all picophytoplankton, using all pooled data (all *p* < 0.001; Figure S7). This result suggests that the growth of heterotrophic bacterioplankton may be associated with autochthonous organic carbon derived from picophytoplankton in the Kuroshio [15,30]; similar results have also been observed in other marine environments [44].

## 3. Materials and Methods

### 3.1. Study Area and Sampling

To understand the dynamics of plankton CR and POC in the Kuroshio ecosystem, eight stations (K101–K108) along a transect (KTV1) between 121.72° and 123° E at 23.75° N were repeatedly surveyed across the Kuroshio east of Taiwan (Figure 1). Samples were collected from eight shipboard measurements taken from the R/V *Ocean Researcher I* between April 2014 and November 2015, which covered the entire season: April, July, September, and November 2014 and March, June, September, and November 2015. At each station, the bottom depth was below 2000 m, and seawater was sampled at eight depths (2–5, 10, 30, 50, 100, 150, 200, and 250 m) using Teflon-coated Go-Flo bottles (20 L, General Oceanics Inc., USA) mounted on a General Oceanic rosette assembly. Subsamples from each sampling depth were taken for the analysis of POC, dissolved inorganic nutrients (e.g., nitrate [NO_3_^−^; N] and phosphate [PO_4_^3−^; P]), chlorophyll *a* (Chl *a*), autotrophic picoplankton (e.g., *Synechococcus*, *Prochlorococcus*, and picoeukaryotes), heterotrophic bacterioplankton (only for 2015 cruises) abundance, and plankton CR, as described below. Please refer to Chen et al. [15,29,44] for a detailed description of the methods used to collect the hydrographic data and analyze the aforementioned response variables.

### 3.2. Physical and Chemical Hydrographics

Temperature and salinity were recorded to a depth of 250 m in the water column using a SeaBird CTD (SBE 9/11 puls, SBE Inc., Barre, VT, USA). Photosynthetically active radiation (PAR) was also measured to a depth of 250 m in the water column using an irradiance sensor (4π; QSP-200L). The depth of the euphotic zone (Z_E_) was taken as the penetration depth of 1% of the surface light. The depth of the mixed layer (M_D_) was based on the potential density criterion of 0.125 kg m^−3^ [45].

Water samples were collected for dissolved inorganic nitrate and phosphate analysis and stored in 100 mL polypropylene bottles. The samples were immediately frozen in liquid nitrogen. A custom-made flow-injection analyzer was used for the nitrate and phosphate analyses, which were characterized by detection limits of 0.3 and 0.01 μM, respectively [46].

The water samples (2 L) for POC analysis were immediately filtered through a Whatman 25 mm GF/F filter (pore size = 0.7 μm), wrapped in aluminum foil, and stored at −20 °C until POC analysis. Both the filter and the aluminum foil had been pre-baked at 500 °C for 2 h. After fuming the filters with HCl, the POC on the filters was measured using an elemental analyzer (Elementa, Vario EL-III, Hanau, Germany) [47].

### 3.3. Biological Variables

The water samples (2L) taken for Chl *a* analysis were immediately filtered through GF/F filter paper (Whatman, 47 mm; pore size = 0.7 µm) and stored in liquid nitrogen. The Chl *a* retained on the GF/F filters was quantified fluorometrically (Turner Design 10-AU-005), as in Parsons et al. [48]. If applicable, for a continuous profile, fluorescence was measured with a fluorometer (AquaTracka III, Chelsea Technologies Group Ltd., Leeds, UK) attached to the SeaBird CTD and calibrated by the measured Chl *a* concentration. Notably, the linear relationships were statistically significant between the measured Chl *a* and the fluorescence values for each sampling period (all *p* ≤ 0.001; data not shown). When applicable, Chl *a* was converted to carbon units using a C–Chl ratio of 65.9, which was estimated from the integrated value over the upper 200 m of the KW [49].

Water samples for picoplankton enumeration were fixed in seawater-buffered paraformaldehyde at a final concentration of 0.2% (*w*/*v*) in the dark [50]; they were subsequently frozen and stored in liquid nitrogen until further analysis. They were then identified and enumerated using a flow cytometer (FACSAria, Becton-Dickinson, East Rutherford, NJ, USA). The different picophytoplankton was distinguished based on cell size (forward- and side-scattering) and autofluorescence in the ranges of orange from phycoerythrin (575 ± 15 nm, for *Synechococcus*) and red from divinyl chlorophyll (>670 nm, for *Prochlorococcus* and photosynthetic picoeukaryotes) under excitation at 488 nm [51]. The heterotrophic bacterioplankton was stained with the nucleic acid-specific dye SYBR^®^ Green I (emission = 530 ± 30 nm) in a 10^4^-fold-diluted commercial solution (Molecular Probes, Oregon, USA) [52]. Known numbers of fluorescent beads (TruCOUNT Tubes, Becton-Dickinson) were simultaneously used to calculate the original cell abundance in each sample [51,53]. Where applicable, cell abundances were converted to carbon units using factors widely applied, including 53 fg C cell^−1^ for *Prochlorococcus*, 250 fg C cell^−1^ for *Synechococcus*, 2100 fg C cell^−1^ for picoeukaryotes, and 20 fg C cell^−1^ for heterotrophic bacterioplankton [42,54,55,56,57]. It should be noted that data for heterotrophic bacterioplankton were only available for 2015 cruises.

The plankton CR was calculated as the decrease in dissolved oxygen (O_2_) during dark incubation from three initial and three dark treatment samples [58]. The treatment samples were siphoned into 350-mL biological oxygen demand (BOD) bottles and incubated for 48 h in a dark chamber filled with running surface water. The concentration of O_2_ was measured using the direct spectrophotometry method [59]. The precision for initial samples was <0.01 mg L^−1^. The difference in O_2_ concentration between the initial and the dark treatment was used to compute the CR. A respiration quotient of 1 was assumed in order to convert respiration from oxygen units to carbon units [48,60].

### 3.4. The Depth-Normalized Integrated Values and Statistical Analysis

Chen et al. [15] showed that the Z_E_ is consistent with an average depth of 103.2 (±12.3) m in this study region. For systematic comparison, the depth-normalized integrated value of chemical and biological variables was therefore integrated over the upper 100 m water depth, and it was estimated using the trapezoidal method for each station. The temporal mean values of depth-normalized integrated variables were averaged over all the stations of the KTV1 transect for each sampling period; the spatial mean values of the depth-normalized integrated variables were averaged over each station of the KTV1 transect for all sampling periods. The depth-normalized integrated and its mean values were applied for further comparison and analysis. SigmaStat (ver. 3.5, Systat Software, Inc., San Jose, CA, USA) was used for simple and multiple linear regression analyses and for analysis of variance (ANOVA).

## 4. Conclusions

The Kuroshio is the western boundary current in the North Pacific Ocean and is the counterpart of the Gulf Stream in the North Atlantic Ocean. The Kuroshio Current transports an enormous volume of materials northwards; however, related studies on organic carbon production and consumption are rare. To explore this, eight shipboard measurements were surveyed between Aril 2014 and November 2015 across the Kuroshio at the KTV1 transect east of Taiwan. Vertically, the seawater temperature displayed a coastal uplift (i.e., onshore lifting and offshore deepening) of the isotherms along the KTV1 transect. Corresponding to the isotherms uplifting, the nutrient (e.g., nitrate and phosphate) concentration contours also demonstrated a coastal uplift pattern along the KTV1 transect. The uplift nutrients may thereafter enhance phytoplankton growth; as expected, higher phytoplankton biomass and subsurface Chl *a* maximum located shallower were observed in the onshore stations. Further analysis showed that phytoplankton was mostly dominated by picophytoplankton, including *Prochlorococcus* (4.91 ±3.10 μg C L^−1^), *Synechococcus* (4.86 ±2.79 μg C L^−1^), and picoeukaryotes (6.74 ±3.00 μg C L^−1^). In the oligotrophic ocean, POC is normally treated as the proxy of phytoplankton. This assumption can be indirectly evidenced by the significantly linear relationship between concentrations of POC and Chl *a*, and the biomass (in carbon unit) ratio of Chl *a* to POC was 102.6% (±83.5) in this study. The mean depth-normalized integrated value (i.e., the upper 100 m water depth) of POC ranged from 12.7 (±2.3) to 21.6 (±5.2) μg C L^−1^ over each sampling period. It also demonstrated a distinct distribution pattern; for example, the mean seasonal values were higher in the summer and autumn, and spatially, its values were higher in the onshore stations than in the offshore stations. To consume organic carbon (plankton CR), the mean depth-normalized integrated rate ranged from 7.07 (±4.57) to 22.27 (±13.76) mg C m^−3^ d^−1^ over each sampling period. Seasonally, there was no distinct pattern observed in the plankton CR. Spatially, the higher rate was found in the onshore station, and it may be attributed to or associated with higher concentrations of phytoplankton or POC in the onshore stations. Further analyses suggested that plankton CR is mostly contributed to by auto- and hetero-picoplankton such as *Prochlorococcus*, *Synechococcus*, picoeukaryotes, and heterotrophic bacterioplankton. Although other planktons such as protozoan and zooplankton were not measured in this study, all results suggest that the variations in plankton CR and POC may be mostly associated with the dynamics of picoplankton in the Kuroshio east of Taiwan. For a comprehensive understanding of organic carbon production and consumption in the Kuroshio, more studies on this related issue are required.

## Data Availability

The dataset for this research is available in the following in-text data citation: doi:10.5061/dryad.p5hqbzksb.

## Supplementary Materials

The following supporting information can be downloaded at: https://www.mdpi.com/article/10.3390/plants11212909/s1, Table S1: Spatial variation of the mixed layer depth and euphotic zone; Figure S1: Temporal variation of temperature and salinity of surface water; Figure S2: Relationship between nitrate and phosphate; Figure S3: Vertical depth profile of mean plankton community respiration at different stations; Figure S4: Vertical depth profile of mean particulate organic carbon concentration; Figure S5: Relationships between concentrations of chlorophyll *a* and picophytoplankton; Figure S6: (a) Temporal and (b) spatial variations of the mean bacterioplankton biomass; Figure S7: Relationships between biomass of heterotrophic bacterioplankton versus biomass of (a) *Prochlorococcus,* (b) picoeukaryotes, and (c) picophytoplankton.

## References

[1] Jahnke R.A. (1996). The global ocean flux of particulate organic carbon: Areal distribution and magnitude. Glob. Biogeochem. Cycles.

[2] Longhurst A. (1995). Seasonal cycles of pelagic production and consumption. Prog. Oceanogr..

[3] Volkman J.K., Tanoue E. (2002). Chemical and biological studies of particulate organic matter in the ocean. J. Oceanogr..

[4] Hedges J.I., Keil R.G., Benner R. (1997). What happens to terrestrial organic matter in the ocean?. Org. Geochem..

[5] Ducklow H.W., Steinberg D.K., Buesseler K.O. (2001). Upper ocean carbon export and the biological pump. Oceanography.

[6] Thomsen L., Aguzzi J., Costa C., De Leo F., Ogston A., Purser A. (2017). The oceanic biological pump: Rapid carbon transfer to depth at continental margins during winter. Sci Rep-Uk.

[7] Alldredge A.L., Silver M.W. (1988). Characteristics, dynamics and significance of marine snow. Progress in Oceanography. Prog. Oceanogr..

[8] Duarte C.M., Agustí S. (1998). The CO_2_ balance of unproductive aquatic ecosystems. Science.

[9] Robinson C., Williams P.J.l.B., del Giorgio P.A., Williams P.J.l.B. (2005). Respiration and its measurement in surface marine waters. Respiration in Aquatic Ecosystems.

[10] Nitani H., Stommel H., Yoshida K. (1972). Beginning of the Kuroshio. Kuroshio: Its Physical Aspects.

[11] Guo Y.J., Barnes M. (1991). The Kuroshio, Part II: Primary productivity and phytoplankton. Oceanography Marine Biological Annual Review.

[12] Madigan D.J., Chiang W.-C., Wallsgrove N.J., Popp B.N., Kitagawa T., Choy C.A., Tallmon J., Ahmed N., Fisher N.S., Sun C.-l. (2016). Intrinsic tracers reveal recent foraging ecology of giant Pacific bluefin tuna at their primary spawning grounds. Mar. Ecol. Prog. Ser..

[13] Liu K.-M., Cheng Y.-T., Chen W.-K., Chen C.-T., Su W.-C. (2008). Examining the density-dependent effect on the growth of dolphinfish, Corphaena hippurus in the Eastern Taiwan waters. J. Fish. Soc. Taiwan.

[14] Saito H., Nagai T., Saito H., Suzuki K., Takahashi M. (2019). Its recognition, scientific activities and emerging issues. Kuroshio Current: Physical, Biogeochemical, and Ecosystem Dynamics.

[15] Chen C.-C., Lu C.-Y., Jan S., Hsieh C.-H., Chung C.-C. (2022). Effects of the coastal uplift on the Kuroshio ecosystem, eastern Taiwan, the western boundary current of the North Pacific Ocean. Front. Mar. Sci..

[16] Chen C.-C., Hsu S.-C., Jan S., Gong G.-C. (2015). Episodic events imposed on the seasonal nutrient dynamics of an upwelling system off northeastern Taiwan. J. Mar. Syst..

[17] Nagai T., Durán G.S., Otero D.A., Mori Y., Yoshie N., Ohgi K., Hasegawa D., Nishina A., Kobari T. (2019). How the Kuroshio Current delivers nutrients to sunlit layers on the continental shelves with aid of near-internal waves and turbulence. Geophys. Res. Lett..

[18] Acabado C.S., Cheng Y.-H., Chang M.-H., Chen C.-C. (2021). Vertical nitrate flux induced by Kelvin-Helmholtz billows over a seamount in the Kuroshio. Front. Mar. Sci..

[19] Richardson T.L., Jackson G.A. (2007). Small phytoplankton and carbon export from the surface ocean. Science.

[20] Hung J.J., Lin P.L., Liu K.K. (2000). Dissolved and particulate organic carbon in the southern East China Sea. Cont. Shelf Res..

[21] Chen D., Zeng L., Boot K., Liu Q. (2022). Satellite observed spatial and temporal variabilities of particulate organic carbon in the East China Sea. Remote Sens..

[22] Huang Y., Chen B., Huang B., Zhou H., Yuan Y. (2019). Potential overestimation of community respiration in the western Pacific boundary ocean: What causes the putative net heterotrophy in oligotrophic systems?. Limnol. Oceanogr..

[23] Huang Y., Laws E., Chen B., Huang B. (2019). Stimulation of heterotrophic and autotrophic metabolism in the mixing zone of the Kuroshio Current and northern South China Sea: Implications for export production. J. Geophys. Res. Biogeosci..

[24] Waku M., Furuya K. (1998). Primary production and community respiration in a warm streamer associated with Kuroshio warm core ring in Spring. J. Oceanogr..

[25] Roughan M., Middleton J.H. (2002). A comparison of observed upwelling mechanisms off the east coast of Australia. Cont. Shelf Res..

[26] Rochford D.J. (1984). Nitrates in eastern Australian coastal waters. Aust. J. Mar. Freshw. Res..

[27] Kobari T., Honma T., Hasegawa D., Yoshie N., Tsutsumi E., Matsuno T., Nagai T., Kanayama T., Karu F., Suzuki K. (2020). Phytoplankton growth and consumption by microzooplankton stimulated by turbulent nitrate flux suggest rapid trophic transfer in the oligotrophic Kuroshio. Biogeosciences.

[28] Jan S., Yang Y.J., Wang J., Mensah V., Kuo T.-H., Chiou M.-D., Chern C.-S., Chang M.-H., Chien H. (2015). Large variability of the Kuroshio at 23.75°N east of Taiwan. J. Geophys. Res. Ocean..

[29] Chen C.-C., Jan S., Kuo T.H., Li S.Y. (2017). Nutrient flux and transport by the Kuroshio east of Taiwan. J. Mar. Syst..

[30] Lai C.-C., Wu C.-R., Chuang C.-Y., Tai J.-H., Lee K.-Y., Kuo H.-Y., Shiah F.-K. (2021). Phytoplankton and bacterial responses to monsoon-driven water masses mixing in the Kuroshio off the east coast of Taiwan. Front. Mar. Sci..

[31] Kodama T., Shimizu Y., Ichikawa T., Hiroe Y., Kusaka A., Morita H., Shimizu M., Hidaka K. (2014). Seasonal and spatial contrast in the surface layer nutrient content around the Kuroshio along 138°E, observed between 2002 and 2013. J. Oceanogr..

[32] Chen C.-T.A., Liu C.T., Pai S.C. (1995). Variations in oxygen, nutrient and carbonate fluxes of the Kuroshio Current. La mer.

[33] Liu K., Suzuki K., Chen B., Liu H. (2021). Are temperature sensitivities of *Prochlorococcus* and *Synechococcus* impacted by nutrient availability in the subtropical northwest Pacific?. Limnol. Oceanogr..

[34] Agawin N.S., Duarte C., Agustí S., Vaqué D. (2004). Effect of N:P ratios on response of Mediterranean picophytoplankton to experimental nutrient inputs. Aquat. Microb. Ecol.

[35] Zhu Z.Y., Zhang J., Wu Y., Lin J. (2006). Bulk particulate organic carbon in the East China Sea: Tidal influence and bottom transport. Prog. Oceanogr..

[36] Shiah F.K., Chung S.W., Kao S.J., Gong G.C., Liu K.K. (2000). Biological and hydrographical responses to tropical cyclones (typhoons) in the continental shelf of the Taiwan Strait. Cont. Shelf Res..

[37] Qu B., Song J., Yuan H., Li X., Li N. (2018). Carbon chemistry in the mainstream of Kuroshio Current in eastern Taiwan and its transport of carbon into the East China Sea shelf. Sustainability.

[38] Liu Q., Kandasamy S., Wang H., Wang L., Lin B., Gao A., Chen C.T.A. (2019). Impact of hydrological conditions on the biogeochemical dynamics of suspended particulate organic matter in the upper mixed layer of the southern East China Sea. J. Geophys. Res. Ocean..

[39] Chen C.-C., Gong G.C., Shiah F.K., Chou W.C., Hung C.C. (2013). The large variation in organic carbon consumption in spring in the East China Sea. Biogeosciences.

[40] Zhong Q., Yu T., Lin H., Lin J., Ji J., Ni J., Du J., Huang D. (2021). 210Po-210Pb disequilibrium in the Western North Pacific Ocean: Particle cycling and POC export. Front. Mar. Sci..

[41] Marañón E., Holligan P.M., Barciela R., González N., Mouriño B., Pazó M.J., Varela M. (2001). Patterns of phytoplankton size structure and productivity in contrasting open-ocean environments. Mar. Ecol. Prog. Ser..

[42] Buck K.R., Chavez F.P., Campbell L. (1996). Basin-wide distributions of living carbon components and the inverted trophic pyramid of the central gyre of the north Atlantic Ocean, summer 1993. Aquat. Microb. Ecol.

[43] Mouriño-Carballido B., Hojas E., Cermeño P., Chouciño P., Fernández-Castro B., Latasa M., Marañón E., Morán X.A.G., Vidal M. (2016). Nutrient supply controls picoplankton community structure during three contrasting seasons in the northwestern Mediterranean Sea. Mar. Ecol. Prog. Ser..

[44] Chen C.-C., Gong G.-C., Chiang K.-P., Shiah F.-K., Chung C.-C., Hung C.-C. (2021). Scaling effects of a eutrophic river plume on organic carbon consumption. Limnol. Oceanogr..

[45] Levitus S. (1982). Climatological Atlas of the Word Ocean, NOAA Professional Paper No. 13.

[46] Gong G.-C., Wen Y.-H., Wang B.-W., Liu G.-J. (2003). Seasonal variation of chlorophyll *a* concentration, primary production and environmental conditions in the subtropical East China Sea. Deep-Sea Res. II Top. Stud. Oceanogr..

[47] Hung C.C., Gong G.C., Chou W.C., Chung C.C., Lee M.A., Chang Y., Chen H.Y., Huang S.J., Yang Y., Yang W.R. (2010). The effect of typhoon on particulate organic carbon flux in the southern East China Sea. Biogeosciences.

[48] Parsons T.R., Maita Y., Lalli C.M. (1984). A Manual of Chemical and Biological Methods for Seawater Analysis.

[49] Furuya K. (1990). Subsurface chlorophyll maximum in the tropical and subtropical western Pacific Ocean: Vertical profiles of phytoplankton biomass and its relationship with chlorophyll a and particulate organic carbon. Mar. Biol..

[50] Liu H., Nolla H.A., Campbell L. (1997). *Prochlorococcus* growth rate and contribution to primary production in the equatorial and subtropical North Pacific Ocean. Aquat. Microb. Ecol.

[51] Chan Y.F., Chung C.C., Gong G.C., Hsu C.W. (2020). Spatial variation of abundant picoeukaryotes in the subtropical Kuroshio Current in winter. Mar. Ecol..

[52] Liu H.B., Suzukil K., Minami C., Saino T., Watanabe M. (2002). Picoplankton community structure in the subarctic Pacific Ocean and the Bering Sea during summer 1999. Mar. Ecol. Prog. Ser..

[53] Chung C.-C., Gong G.-C. (2019). Attribution of the growth of a distinct population of *Synechococcus* to the coverage of lateral water on an upwelling. Terr. Atmos. Ocean. Sci..

[54] Morel A., Ahn Y.-H., Partensky F., Vaulot D., Claustre H. (1993). *Prochlorococcus* and *Synechococcus*: A comparative study of their optical properties in relation to their size and pigmentation. J. Mar. Res..

[55] Campbell L., Nolla H.A., Vaulot D. (1994). The importance of *Prochlorococcus* to community structure in the central North Pacific Ocean. Limnol. Oceanogr..

[56] Lee S., Fuhrman J.A. (1987). Relationship between biovolume and biomass of naturally derived marine bacterioplankton. Appl. Environ. Microbiol..

[57] Kana T.M., Glibert P.M. (1987). Effect of irradiances up to 2000 μE m^−2^ s^−1^ on marine *Synechococcus* WH7803—II. Photosynthetic responses and mechanisms. Deep. Sea Res. Part A. Oceanogr. Res. Pap..

[58] Gaarder T., Grann H.H. (1927). Investigations of the production of plankton in the Oslo Fjord. Rapport et Proces-Verbaux des Reunions. Cons. Perm. Int. Pour L’explor. Mer.

[59] Pai S.-C., Gong G.-C., Liu K.-K. (1993). Determination of dissolved oxygen in seawater by direct spectrophotometry of total iodine. Mar. Chem..

[60] Hopkinson C.S. (1985). Shallow-water benthic and pelagic metabolism: Evidence of heterotrophy in the nearshore Georgia Bight. Mar. Biol..

