# Supplementary material for: Plankton Community Respiration and Particulate Organic Carbon in the Kuroshio East of Taiwan
Source: Plants (Basel). 2022 Oct 29;11(21):2909. doi: 10.3390/plants11212909 (PMC9655403; doi:10.3390/plants11212909)
Supplement: Supplementary file 1 [file plants-11-02909-s001.zip › plants-1907510-supplementary.pdf]

Supporting Information for

Plankton community respiration and particulate organic carbon in the Kuroshio east of Taiwan

Chung-Chi Chen[1,2]*, Pei-Jie Meng[2,3,4]*, Chih-hao Hsieh[5], Sen Jan[5]

[1]Department of Life Science, National Taiwan Normal University
88, Sec. 4, Ting-Chou Road
Taipei 11677, Taiwan

[2]Graduate Institute of Marine Biology, National Dong Hwa University
Checheng, Pingtung 94450, Taiwan

[3]General Education Center, National Dong Hwa University
Shoufeng, Hualien 97401, Taiwan

[4]National Museum of Marine Biology and Aquarium
Checheng, Pingtung 94450, Taiwan

[5]Institute of Oceanography, National Taiwan University
Taipei 10617, Taiwan

Running title: Plankton community respiration and POC in the Kuroshio

*Corresponding author:
E-mail: ccchen@ntnu.edu.tw (C.-C.C); pjmeng@gms.ndhu.edu.tw (P.-J.M.)
Phone: 886-2-2930-2275 (C.-C.C)
Fax # : 886-2-2931-2904 (C.-C.C)

Contents of this file: Supplementary Table S1 and figures S1–S7

Introduction

This supporting information provides the supplemental table and figures cited in the article.

Table S1. Spatial variations of the mean (± standard deviations) values (m) of the mixed layer depth (M_D) and the euphotic zone (Z_E) averaged across each station of the KTV1 transect for all sampling periods.

Stations	M_D	Z_E
k101	29.4 (± 26.8)	88.3 (± 13.2)
k102	20.5 (± 21.6)	98.2
k103	17.9 (± 12.9)	-
k104	38.8 (± 37.5)	105.9 (± 11.9)
k105	40.8 (± 37.3)	108.2 (± 11.7)
k106	54.5 (± 35.4)	108.2 (± 7.8)
k107	54.4 (± 48.6)	122.7
k108	49.3 (± 47.2)	106.8 (± 7.3)

-: No data

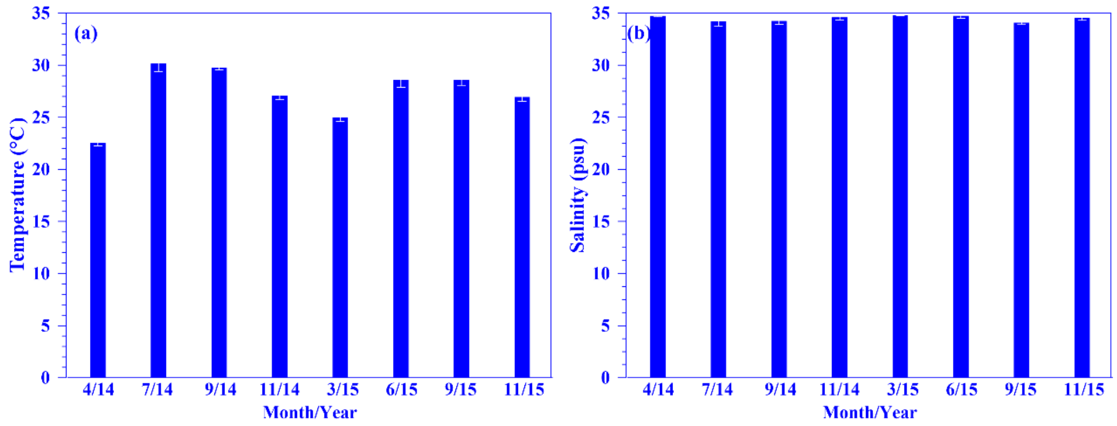

Figure S1: Temporal variations of the mean values of (**a**) temperature and (**b**) salinity of surface water averaged across all stations of the KTV1 transect over each sampling period. The standard deviations are illustrated as vertical white lines with caps.

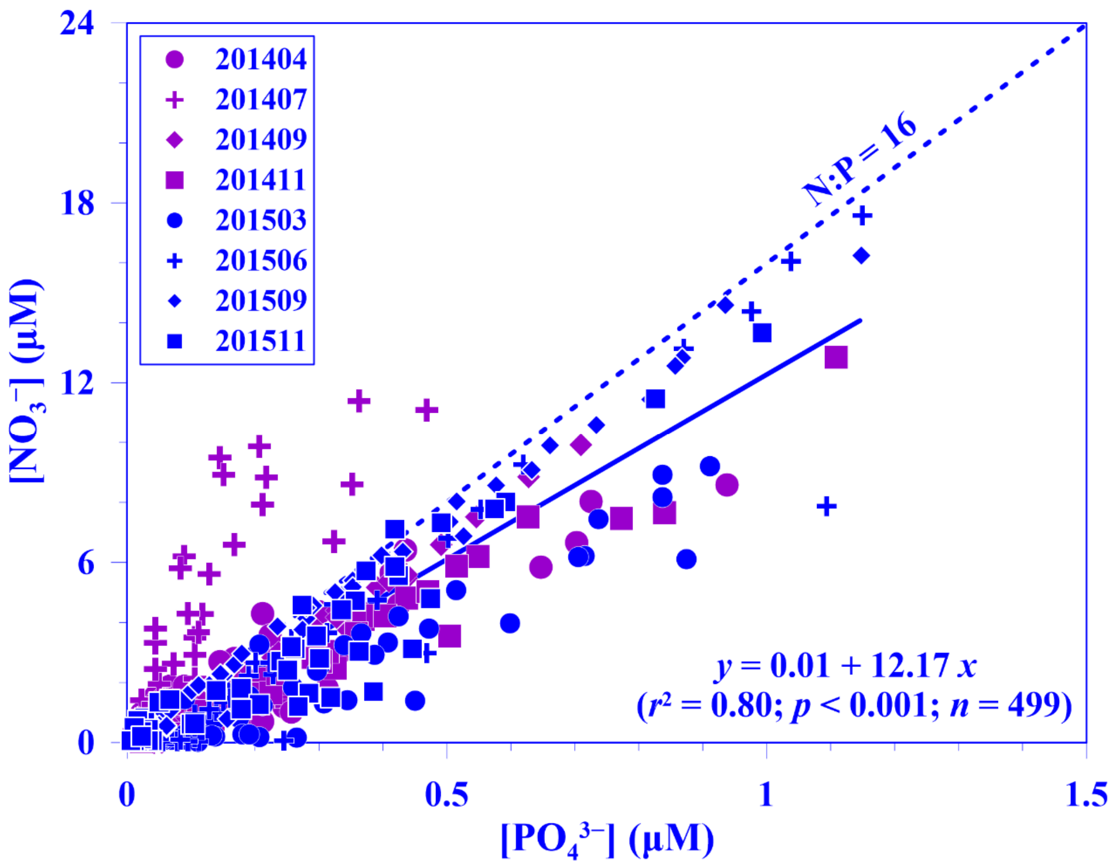

Figure S2: Relationship between nitrate (NO_3^-) and phosphate (PO_4^{3-}) using all measured data at different sampling periods, as indicated by the different symbols and colors in the panel, in this study. For guidance, the N/P = 16 line (dashed line) is shown.

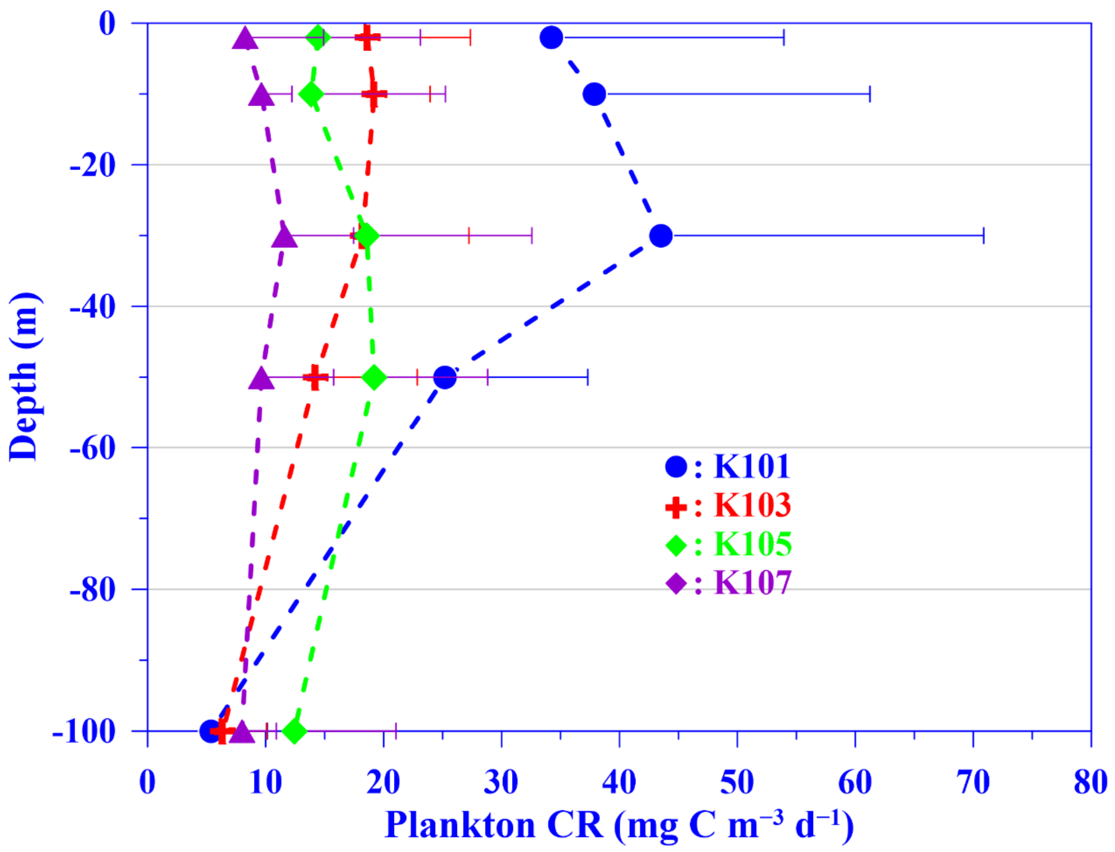

Figure S3: Vertical depth profile of mean plankton community respiration (plankton CR) at different sampling stations as symbols indicate in the panel. The mean values are averaged for each sampling depth at each sampling stations over all sampling periods. The standard deviations are illustrated as positive horizontal lines with caps.

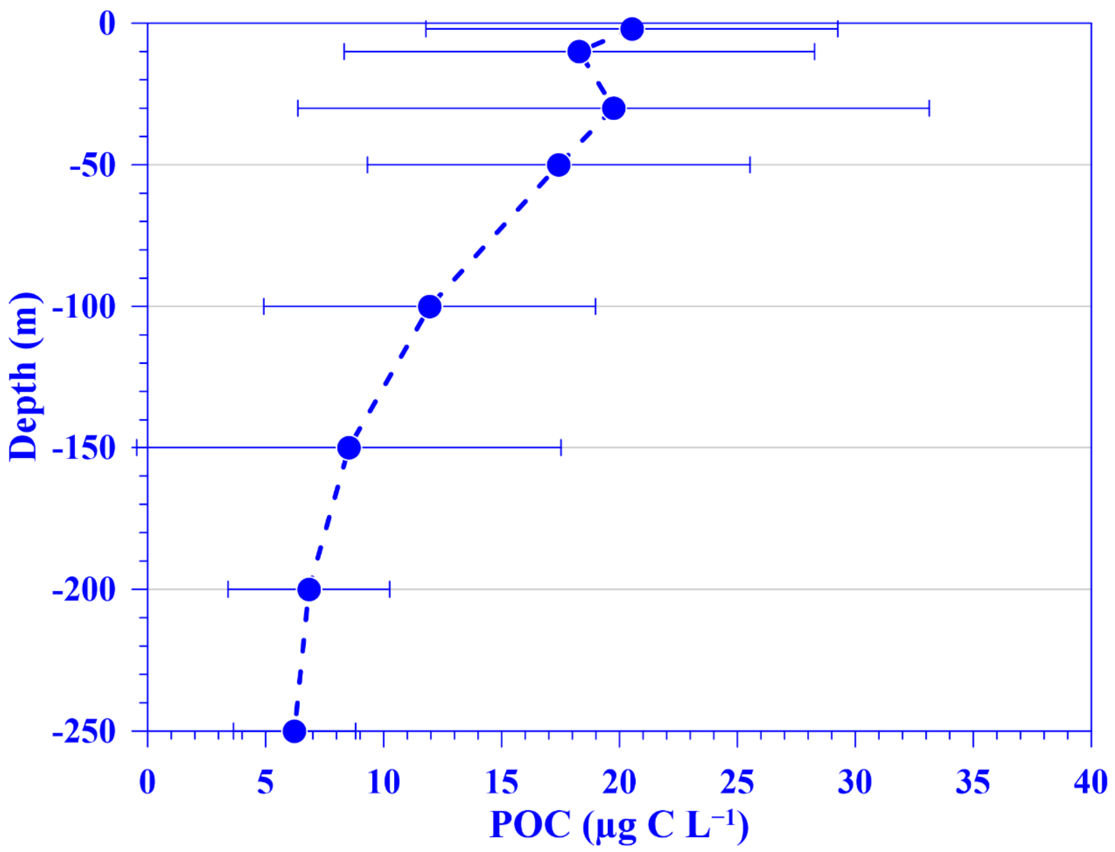

Figure S4: Vertical depth profile of mean particulate organic carbon (POC) concentration across the KTV1 transect in the Kuroshio. The mean values are averaged for each sampling depth of all sampling stations and periods. The standard deviations are illustrated as horizontal lines with caps.

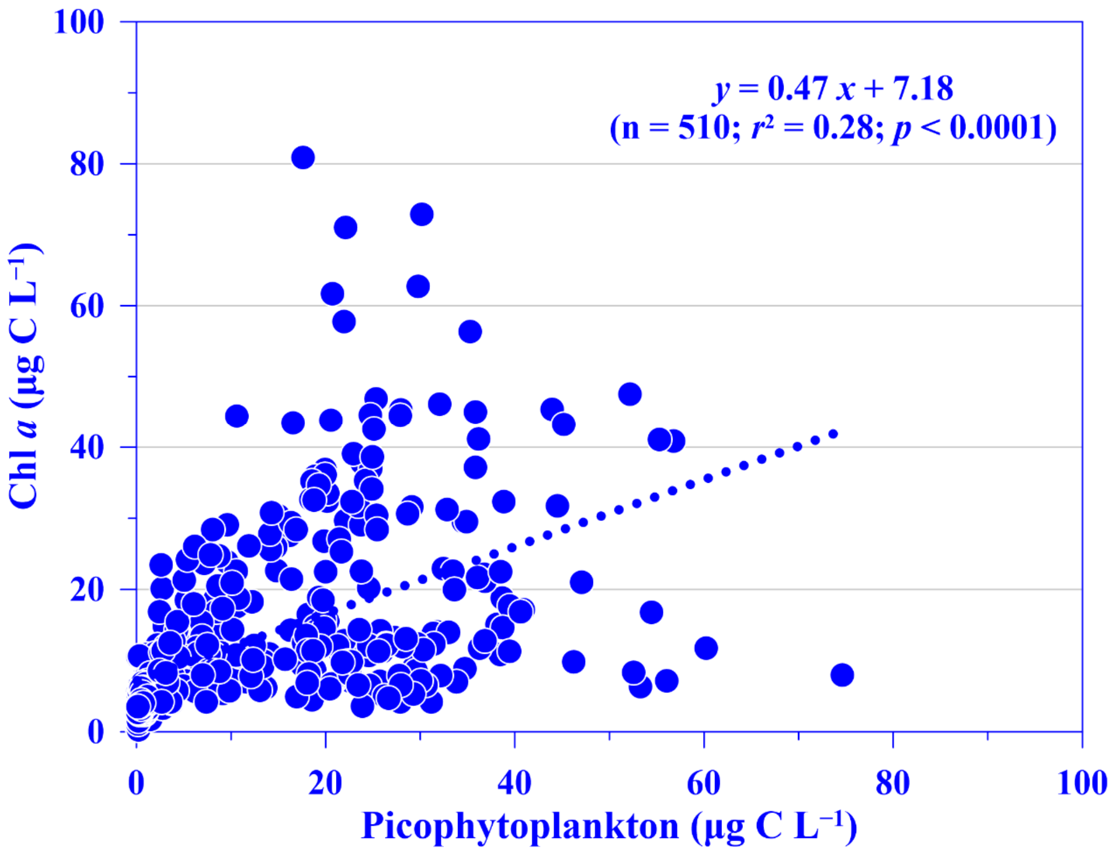

Figure S5: Relationship between the concentration of chlorophyll *a* (Chl *a*) and picophytoplankton for all pooled data. The r^2 and p values of linear relationship are shown.

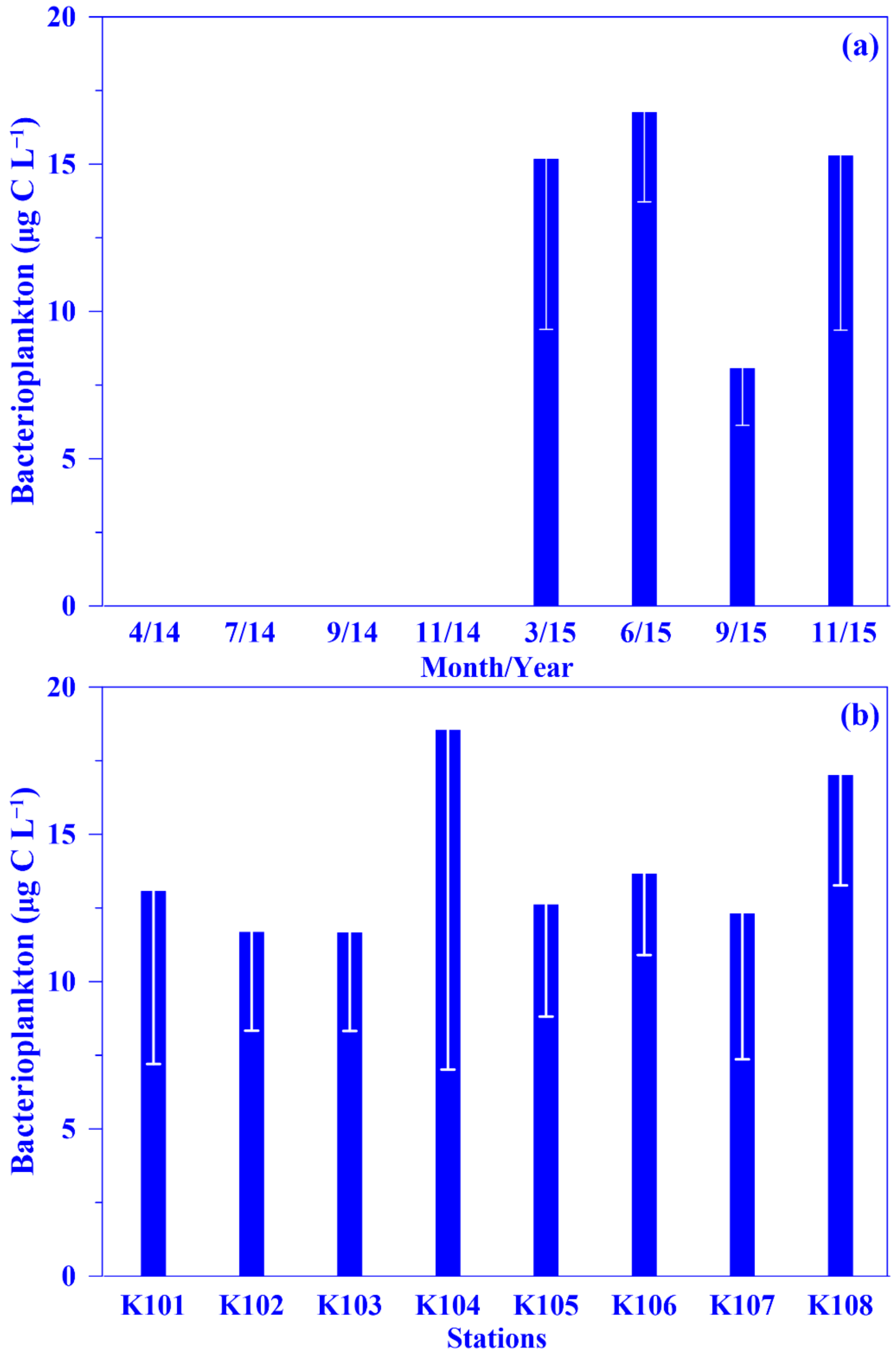

Figure S6: (**a**) Temporal (sampling period) and (**b**) spatial (sampling station) variations of the mean values of heterotrophic bacterioplankton across the KTV1 transect of the Kuroshio. The standard deviations are illustrated as vertical white lines with caps.

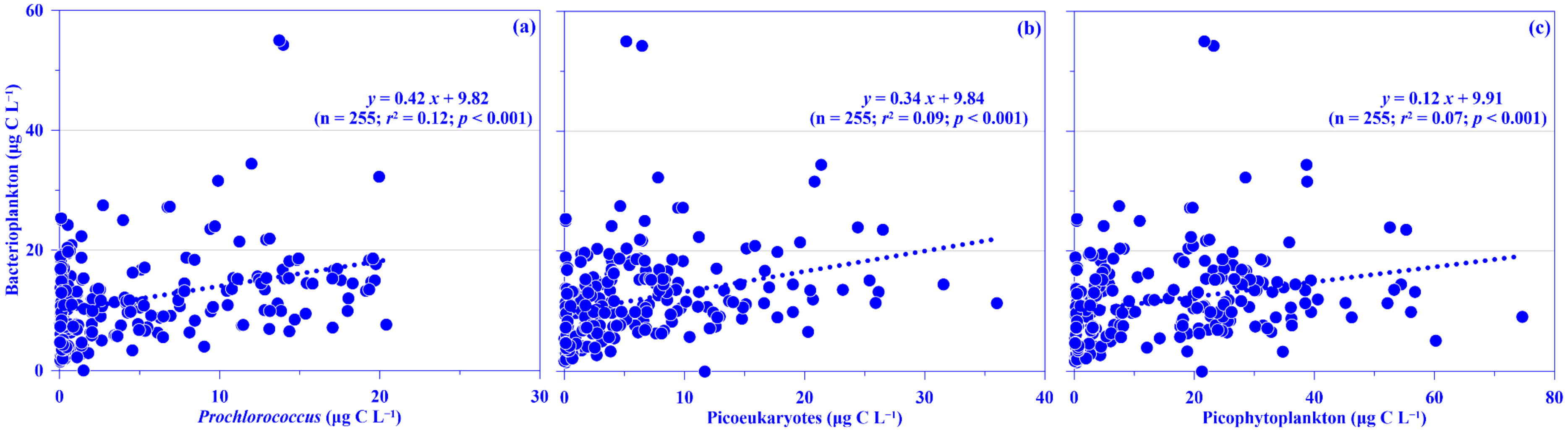

Figure S7: Relationships between biomass of heterotrophic bacterioplankton and the biomass of (**a**) *Prochlorococcus*, (**b**) picoeukaryotes, and (**c**) picophytoplankton for all pooled data. The r^2 and p values of linear relationships are shown.
